# The Predictive Validity of Savry Ratings for Assessing Youth Offenders in Singapore: A Comparison With YLS/CMI Ratings

**DOI:** 10.1177/0093854815616842

**Published:** 2015-12-09

**Authors:** Chi Meng Chu, Mui Leng Goh, Dominic Chong

**Affiliations:** Ministry of Social and Family Development, Singapore

**Keywords:** protective factors, recidivism, risk assessment, SAVRY, violence, juvenile offenders

## Abstract

Empirical support for the usage of the SAVRY has been reported in studies conducted in many Western contexts, but not in a Singaporean context. This study compared the predictive validity of the SAVRY ratings for violent and general recidivism against the Youth Level of Service/Case Management Inventory (YLS/CMI) ratings within the Singaporean context. Using a sample of 165 male young offenders (*M_follow-up_* = 4.54 years), results showed that the SAVRY Total Score and Summary Risk Rating, as well as YLS/CMI Total Score and Overall Risk Rating, predicted violent and general recidivism. SAVRY Protective Total Score was only significantly predictive of desistance from general recidivism, and did not show incremental predictive validity for violent and general recidivism over the SAVRY Total Score. Overall, the results suggest that the SAVRY is suited (to varying degrees) for assessing the risk of violent and general recidivism in young offenders within the Singaporean context, but might not be better than the YLS/CMI.

Youth violence leads to large costs to victims and government. The estimated cost of homicides and assault-related injuries among youth in the United States is $16.2 billion—in terms of lifetime combined medical and work loss costs (Centers for Disease Control and Prevention, 2015). Considering the high stakes to the youth and also to public safety, accurate and comprehensive risk assessment procedures play a critical part in the juvenile justice system. Specifically, risk assessment procedures assist in identifying the level of intervention needs for each offender and making decisions regarding placements, supervision levels, case management planning, and treatment, with the ultimate aim of preventing future reoffending (Andrews & Bonta, 2010; Lodewijks, Doreleijers, & de Ruiter, 2008).

## Assessing Risk of Violent and General Recidivism in Youth Offenders

Research studies have shown that structured risk assessment methods (i.e., actuarial and structured clinical judgment approaches) are not only more accurate than unstructured clinical judgment, but they are also preferred because of their increased transparency and reliability (see Heilbrun, Yasuhara, & Shah, 2010, for a review). Although it is efficient, highly flexible, and allows clinicians to focus on case-specific information and violence prevention, unstructured clinical judgment has been criticized for being subjective, unreliable, poorly validated, and unable to detail the decision-making process (Monahan & Steadman, 1994; Quinsey, Harris, Rice, & Cormier, 2006; Webster, Douglas, Eaves, & Hart, 1997). However, purely actuarial and structured clinical judgment approaches have been found to be moderately predictive of violence (Ægisdóttir et al., 2006; de Vogel, de Ruiter, van Beek, & Mead, 2004; Singh, Grann, & Fazel, 2011).

In the area of youth risk assessment, the Structured Assessment of Violence Risk in Youth (SAVRY; Borum, Bartel, & Forth, 2002, 2006) is a widely used structured risk assessment measure for assessing a youth offender’s risk of violent recidivism. Comprising risk factors that assess historical, social/contextual, and individual/clinical domains, the SAVRY also contains protective factors. In particular, the historical risk factors are commonly viewed as static risk factors that are not amenable to change through planned intervention over time. Hence, these static factors are unlikely to be ameliorated for purpose of managing or reducing the risk of future offending over time (Douglas & Skeem, 2005). However, the social/contextual, individual/clinical, and protective factors can be considered dynamic risk factors for reoffending, which can be changed as a result of deliberate intervention (Webster, Douglas, Belfrage, & Link, 2000), and hence reducing the overall level of risk for future offending. Concomitantly, the Youth Level of Service/Case Management Inventory (YLS/CMI; Hoge & Andrews, 2002, 2011) represents one of the most widely used, structured risk assessment measures for assessing the risk of general recidivism and criminogenic needs in youth offenders. Similar to the SAVRY, the YLS/CMI is composed of static and dynamic risk factors that are associated with reoffending.

Some empirical support for the usage of the SAVRY have been reported in studies conducted in the many Western jurisdictions, which include the United States (e.g., McGowan, Horn, & Mellott, 2011; Area Under Curve [AUC]_Violence_ = .72),^1^ the United Kingdom (e.g., Dolan & Rennie, 2008; AUC_GR_ = .69, AUC_VR_ = .64),^2^ Canada (e.g., Schmidt, Campbell, & Houlding, 2011; AUC_VR_ = .57 − .78, AUC_NVR_ = .63 − .78),^3^ the Netherlands (e.g., Lodewijks, Doreleijers, & de Ruiter, 2008; AUC_VR_ = .65 − .76), Finland (e.g., Gammelgård, Koivisto, Eronen, & Kaltiala-Heino, 2008; AUC_Violence_ = .55 − .84), Spain (e.g., Hilterman, Nicholls, & van Nieuwenhuizen, 2014; AUC_GR_ = .70 − .71, AUC_VR_ = .68 − .75), and Australia (e.g., Shepherd, Luebbers, Ferguson, Ogloff, & Dolan, 2014; AUC_GR_ = .49 − .81, AUC_VR_ = .47 − .76). Currently, there is no published study on the predictive validity of SAVRY risk and protective factor ratings for recidivism in an Asian context.

In comparison, there is a large body of empirical evidence that supports the usage of YLS/CMI across Western and Asian contexts. In particular, these studies have been conducted in the United States (e.g., Onifade et al., 2008; AUC_GR_ = .62), the United Kingdom (e.g., Marshall, Egan, English, & Jones, 2006; AUC_Violence_ = .73 − .75, AUC_No of Charges_ = .73), Canada (e.g., Schmidt et al., 2011; AUC_VR_ = .58 − .65, AUC_NVR_ = .53 − .73), Spain (e.g., Hilterman et al., 2014; AUC_GR_ = .67 − .71, AUC_VR_ = .69 − .73), Australia (e.g., McGrath & Thompson, 2012; AUC_GR_ = .65), Japan (e.g., Takahashi, Mori, & Kroner, 2013; AUC_GR_ = .61 − .87, AUC_VR_ = .50 − .85, AUC_NVR_ = .64 − .87), and Singapore (e.g., Chu et al., 2015; AUC_GR_ = .65 − .67).

In a meta-analytic study on the youth risk assessment measures, Olver, Stockdale, and Wormith (2009) found that the mean-weighted correlation between SAVRY Total Scores and general recidivism was .32 (*k* = 7); and for violent recidivism, the mean-weighted correlation was .30 (*k* = 9). In comparison, the mean-weighted correlation between YLS Total Scores and general recidivism was .32 (*k* = 19), and the mean-weighted correlation between YLS total scores and violent recidivism was .26 (*k* = 9). As a general rule for practice, AUCs greater than .54, .63, and .71, as well as correlation coefficients (*r*) that are greater than .10, .24, and .37, are regarded as small, moderate, and large effects, respectively (Rice & Harris, 2005).

In addition, several studies have shown that the SAVRY Protective Total Score has poor to good predictive validity for desistance from general (AUC = .58 − .91) and violent (AUC = .56 − .77) recidivism—although closer examination revealed that they were cross-cultural differences relating to gender and ethnicity issues (e.g., Dolan & Rennie, 2008; Lodewijks et al., 2008; Rennie & Dolan, 2010; Schmidt et al., 2011; Shepherd et al., 2014). For example, Shepard et al. (2005) found that there were differences between ethnic groups in Australia in terms of predictive validity of the SAVRY protective factor domain for violent and general recidivism whereby the SAVRY ratings yielded moderate to high AUCs for the English Speaking Background and Indigenous subgroups but poor effect sizes for the Culturally and Linguistically Diverse subgroup. Presumably, the differences in the cultural experiences and applicability of the SAVRY items to these culturally diverse groups are central to the differences in performance of the measure.

In their meta-analysis, Olver et al. (2009) also showed that the ratings of the SAVRY and YLS measures had lower predictive validity for violent (for SAVRY: mean-weighted correlation of .26 vs. .34; for YLS measures: mean-weighted correlation of .18 vs. .28) and general recidivism (for SAVRY: mean-weighted correlation of .26 vs. .38; for YLS measures: mean-weighted correlation of .26 vs. .35) when they were used in other Western contexts outside Canada. Olver et al. suggested that “‘international’ differences contributed to the variability across studies” (p. 348). Furthermore, Andrews et al. (2011) postulated that such variability across contexts could be a function of the authors’ allegiance and reliability of outcome measure(s), as well as the differences in legislations, definitions of outcomes, and interpretation of the criteria for rating the risk assessment measure.

The few studies on the YLS/CMI in Asian settings (e.g., Chu et al., 2015; Takahashi et al., 2013) suggest that the predictive validity of the ratings for recidivistic outcomes were lower than those obtained in Canadian contexts, but were similar to the rest of the non-Canadian studies. Taken together, there is a strong rationale to investigate the applicability of youth offender risk assessment measures when adapting them for another jurisdiction.

## The Usage of Youth Risk Assessment Measures in Singapore

Singapore is an independent island-state in South East Asia with a land area of 718 square kilometers and a total population of 5.54 million (Singapore Department of Statistics, 2015). Apropos of crime statistics, youth arrests accounted for about 10% of all arrests in Singapore (Singapore Police Force, 2013). Many statutes in Singapore are based on English common law (e.g., the Criminal Procedure Code, 2012), but there are some statutes that are based on legislation from other jurisdictions (e.g., India), which (nonetheless) are still formulated by the English in 1800s. Therefore, similarities with other jurisdictions exist in the way that offenses are defined in Singapore, but the exact language of the laws might differ. It is important to note that cultures and societies define what attitudes and behaviors are deemed as normal and deviant. Notwithstanding that there is cross-cultural agreement about what constitutes offending behavior, the development of deviant attitudes and behaviors can vary as a result of cultural norms, gender roles, morals, religion, taboos, and expectations (e.g., Bhugra, Popelyuk, & McMullen, 2010; Lahlah, Van der Knaap, Bogaerts, & Lens, 2013). These can lead to differences in motivation, risk factors, and pathways for offending especially when cross-cultural differences as to how individuals cope, self-regulate, or even report crime are also taken into account. For example, there are variations in how individuals express anger across different cultures (Matsumoto, Yoo, & Chung, 2010), and this could lead to differences in the manifestation and perpetuation of violence in situations when provocation happens.

In Singapore, the YLS/CMI (Hoge & Andrews, 2002, 2011) was chosen by the relevant youth justice agencies as the primary risk assessment measure to assess the risk and needs of youth offenders in early to mid-2000s (see Chua, Chu, Yim, Chong, & Teoh, 2014). The utility of the YLS/CMI and its screening version for assessing the risk of recidivism and identifying criminogenic needs in youth offenders was investigated in several studies (e.g., Chu et al., 2015; Chu, Ng, Fong, & Teoh, 2012; Chu, Yu, Lee, & Zeng, 2014; Zeng, Chu, Koh, & Teoh, 2015), and the YLS measures were found to have adequate predictive validity for assessing the risk of violent (AUC_YLS/CMI-SV_Male_ = .62) and general recidivistic outcomes (AUC_YLS/CMI_Male_ = .65; AUC_YLS/CMI_Female_ = .67; AUC_YLS/CMI-SV_Male_ = .65; AUC_YLS/CMI-SV_Female_ = .59) but not sexual recidivism (AUC_YLS/CMI_Male_ = .29). In addition, there was a local study that used the SAVRY to identify criminogenic needs in gang-affiliated and nongang-affiliated youth offenders, and group differences were only found for one risk factor (i.e., peer delinquency) after adjusting for multiple comparisons (Chu, Daffern, Thomas, & Lim, 2011).

Given that the SAVRY is a risk assessment measure that is specifically developed to assess violence risk in youth, there is justification for usage despite the widespread adoption of the validated YLS/CMI within the Singapore context. Such a violence risk assessment measure can presumably provide better resolution on the risk factors for violence and provide important information for the management of violence risk. Nevertheless, there is currently no published study on the predictive validity of the SAVRY ratings for assessing the risk of recidivism in youth offenders in Singapore.

## Present Study

The present study sought to (a) compare the predictive validity of the SAVRY risk ratings for violent and general recidivism in Singapore against YLS/CMI, an established youth risk assessment measure, and (b) examine the predictive validity of the SAVRY protective factor ratings for desistance from violent and general recidivism.

The following hypotheses were formulated for the present study: (a) The SAVRY ratings would have higher predictive validity for violent recidivism than YLS/CMI ratings, and (b) the SAVRY and YLS/CMI ratings would have comparable predictive validity for general recidivism.

## Method

### Definition of Violence and Violent Offenses

This study generally adopted the definition provided in the SAVRY manual, which defined violence as “an act of battery or physical violence that is sufficiently severe to cause injury to another person or persons, regardless of whether the injury occurs . . . or a threat made with a weapon in hand” (Borum et al., 2002, p. 19), but this study excluded sexual violence in its definition of violence. Violence offenses included armed robbery, (physical) assault, attempted murder, causing bodily harm, making threats to harm or kill, murder, rioting, robbery, and unlawful use of weapon.

### Source Sample

The sample comprised 163^4^ youth male offenders (aged 12 to 18 years), who were consecutively referred for psychological evaluation and/or treatment at the Clinical and Forensic Psychology Service (CFPS) of the Ministry of Social and Family Development, Singapore, between January 2004 and December 2005. All youth were charged with and convicted of criminal offenses, and were placed on probation following evaluation and sentencing. The probation services and the courts refer youth offenders to CFPS for a psychological evaluation during pre-sentence assessment when required. Typically, referrals include youth offenders with sexual and violent offending issues, chronic and repetitive offending patterns, and mental health issues. In addition, CFPS also accepts referrals post sentence from the probation services and youth correctional institutions for assessment and treatment of offense-related and/or mental health issues.

#### Sociodemographics

The average age of the sample at time of referral was 15.96 years (*SD* = 1.42, range = 12-18). The majority had received at least secondary-level mainstream education (*n* = 156, 95.7%) and came from an intact family of origin (*n* = 102/161,^5^ 63.4%). The ethnic composition of the sample was 54% Chinese (*n* = 88), 46% non-Chinese (*n* = 75).^6^

#### Offender Characteristics

The average age of the youth offenders at their first charged offense was 15.42 years (*SD* = 1.41, range = 12-18). One fifth (*n* = 33) of the current sample had committed other offenses prior to their index offenses (i.e., those offenses that brought them into contact with CFPS). In terms of index offenses, 36.2% (*n* = 59) had committed violent offenses, 21.5% (*n* = 35) had committed sexual offenses,^7^ 49.7% (*n* = 81) had committed theft or fraud offenses,^8^ and 4.9% (*n* = 8) had committed substance use^9^ offenses. It was also noted that 17.8% (*n* = 29) had a history of weapon use, and 40.5% (*n* = 66) had a history of substance use.

### Measures

#### Savry

The SAVRY (Borum et al., 2002, 2006) is a 24-item risk assessment instrument that was developed from existing research and the professional literature on adolescent development, as well as youth violence. It is based on the structured clinical judgment model (see Webster et al., 1997) and is designed to assist in the assessment and intervention planning for youths (aged 12 to 18 years) where there appears to be a risk of violence. The risk items are classified into three risk domains (Historical, Social/Contextual, and Individual/Clinical), and each risk item is rated on a 3-point scale (Low, Moderate, High) according to specific rating guidelines. For the purpose of the present study, the SAVRY ratings were assigned numerical values “0” to *low*, “1” to *moderate*, and “2” to *high*. The SAVRY Total Score was derived by summing up the individual item scores, and the SAVRY Protective Total Score was derived by summing up the individual protective factor score (Present [1] or Absent [0]). Last, the rater would assign a Summary Risk Rating (SRR) of low, moderate, or high risk of violence after considering all the risk and protective factors present.

#### Yls/Cmi

The YLS/CMI (Hoge & Andrews, 2002, 2011) is a structured assessment instrument designed to facilitate the effective intervention and rehabilitation of youth offenders (aged 12 to 18 years) by assessing their risk level and criminogenic needs. It consists of 42 items divided into eight subscales (Prior/Current Offenses/Dispositions, Family Circumstances/Parenting, Education/Employment, Peer Relations, Substance Abuse, Leisure/Recreation, Personality/Behavior, and Attitudes/Orientation). The item scores (i.e., the number of indicated risk factors/needs) can be aggregated to obtain a total risk/needs score. With regard to the determination of the cutoff scores, the distributions of scores from the original normative samples, the probability of future recidivism for individuals with particular scores, and the specificity and sensitivity of the scores were considered (as advised by Professor Hoge). The cutoff scores of the risk bins for the male youth offenders under community supervision in Singapore are 0 to 10 (*low*), 11 to 19 (*moderate*), 20 to 26 (*high*), and 27 to 42 (*very high*; see Chu et al., 2015). For the purpose of this study, the *high* and *very high* risk bins were collapsed as none of the youth offenders was assessed to be within the *very high* risk bin. Furthermore, the professional override and the YLS/CMI strengths were not coded in this study and hence not included in the analyses.

### Procedure

Approval to conduct this retrospective study of research was granted by the Ministry of Social and Family Development, Singapore. Due to the retrospective nature of the study, informed consent could not be obtained from the participants for the release of information, but the data were deidentified following successful linkage of data sources. Data were collected from multiple data sources, including (a) psychological reports prepared by psychologists at CFPS, (b) pre-sentencing reports prepared by probation officers, (c) charge sheets, (d) statement of facts, (e) any previously existing assessment and treatment reports on the youths’ CFPS files, and (f) school reports. All psychological reports contain specific information pertaining to several key areas of assessment, which includes personal, family, psychiatric, and criminal offending histories, as well as the current offending behaviors and risk management issues.

The following information was collated from the various data sources:

- Sociodemographic characteristics: age at referral, ethnicity (i.e., Chinese vs. non-Chinese), and family structure (i.e., intact vs. non-intact family of origin).
- Offender characteristics: age at first charged offense, type and number of index offenses, past offense history (i.e., previous charged and convicted offenses), history of weapon use, and history of substance use (i.e., alcohol, illicit drugs, and inhalants).
- Recidivism: Recidivism data were obtained on February 4, 2009, following the completion of coding of all other variables. In terms of definition, general recidivism refers to any presence of sexual, violent, or nonviolent offenses that were committed following the initial court order, breaches of court orders, or any combination of the aforementioned outcomes. Violent recidivism refers to the violent offenses (e.g., physical assault, rioting, murder, and robbery) that were committed following the initial court order, whereas nonviolent recidivism refers to the nonviolent nonsexual offenses (e.g., theft, fraud, burglary, drug use, and drug trafficking) that were committed after the initial court order. In addition, sexual recidivism refers to sexual offenses (e.g., indecent exposure, molestation, peeping, rape, and sodomy) that were committed after the initial court order.

For the purpose of this study, the SAVRY and YLS/CMI were coded from case files using the aforementioned materials between November 2008 and January 2009. With regard to the coding of the SAVRY and YLS/CMI, three research assistants and two psychologists received formal training at CFPS to score the SAVRY and YLS/CMI. The raters coded up to 30 randomly selected files for inter-rater reliability purpose, and the intra-class correlation coefficients (ICC; two-way random, single-rater, and absolute agreement) for the SAVRY Total Score, SAVRY SRR, SAVRY Total Protective score, and YLS/CMI Total Score were .67 (good), .88 (excellent), .85 (excellent), and .79 (excellent), respectively (see Cicchetti, 1994, for a classification of ICCs). In addition, the ICCs for the SAVRY Historical, Social/Contextual, and Individual/Clinical subscales were .69 (good), .42 (fair), and .68 (good). Furthermore, the inter-rater agreement for the YLS/CMI subscales was 88% for Prior/Current Offenses/Dispositions, 73.3% for Family Circumstances/Parenting, 82.9% for Education/Employment, 85% for Peer Relations, 88% for Substance Abuse, 80% for Leisure/Recreation, 71.4% for Personality/Behavior, and 92% for Attitudes/Orientation.

Inter-rater reliability were specifically not examined for sociodemographic and offense variables, but the research assistants were aided by a set of detailed coding guidelines and were given a daily group debrief to address any coding difficulties. A SAVRY or YLS/CMI item was not scored if information necessary for coding was unavailable. The relevant risk assessment data for the SAVRY and YLS/CMI were not analyzed if there were more than two and five omitted items, respectively; only two cases had more than two omitted items for the SAVRY (which were deleted from the sample; hence, we have a total of 163 youth). When necessary, the subscale and total scores were subsequently prorated for missing ratings before analyses (prorated score = raw score / [total number of items − number of missing items] × total number of items). All the research assistants were blind to the recidivism data, which were subsequently obtained on February 4, 2009, and coded by first author following the completion of the initial coding of sociodemographic and offense information.

### Statistical Analyses

The sample was characterized using descriptive statistics, with categorical data reported as numbers and percentages, and continuous data presented in relation to the mean and standard deviation. Histograms of the continuous data were plotted to check for skewed distributions. Correlational and Receiver Operating Characteristics (ROC) analyses were conducted to examine the predictive validity of the SAVRY ratings (correlation coefficient and the AUC were reported for each type of rating) during entire follow-up period. To compare the AUCs of ROC curves for SAVRY and YLS/CMI ratings, *z* tests for dependent groups (Hanley & McNeil, 1983) were used to ascertain whether the AUCs differed significantly. Although the SAVRY SRR was rated for the prediction of violent recidivism, its utility for predicting the risk of general recidivism was examined in the present study. Critical ratio *z* is defined as $Z=\left(A_{1}-A_{2}\right)/\sqrt{SE_{1}^{2}+SE_{2}^{2}-2rSE_{1}SE_{2}}$; and *z* values of ≥ |1.96| were taken as evidence that the true areas under the ROC curves were different. Benjamini and Hochberg False Discovery Rate (FDR) corrections (Benjamini & Hochberg, 1995) were also conducted to adjust for Type I error that may arise from multiple comparisons. The FDR correction is designed to control the expected proportion of rejected null hypotheses that were incorrect rejections and is a less conservative but more powerful statistical approach than Bonferroni-type adjustments.

In addition, Cox regression analyses were conducted to compare the general and violent recidivistic outcomes of the offenders with different risk levels to account for the differences in time-at-risk. In particular, the total scores, risk ratings, protective or strengths were entered as single predictors in the Cox regression models. Furthermore, SAVRY subscale scores (i.e., Historical, Social/Contextual, and Individual/Clinical) were simultaneously entered into Cox regression models to examine their unique contributions to predicting violent and general recidivism while accounting for differences in time-at-risk. Last, SAVRY subscale Score and Protective Total Score were entered sequentially as two blocks (i.e., SAVRY Total Scores, then Protective Total Score) in Cox regression models to examine the incremental contribution of the protective factors in predicting both violent and general recidivism. Analyses were carried out using the SPSS Version 19.

## Results

### Recidivism data

The mean follow-up period was 1,658.9 days (*SD* = 298.9, range = 1,127-2,906). With regard to the recidivism rates, 54% (*n* = 88) of the current sample was convicted of new offense(s) during the follow-up period; specifically, 16% (*n* = 26) had committed violent offenses. It was noted that more than a third (*n* = 61, 37.4%) reoffended during their court orders.

### Predictive Validity of the Savry and YLS/CMI Ratings for Recidivistic Outcomes

#### Correlational Analyses

Table 1 shows the correlations between the ratings for the risk assessment measures and recidivistic outcomes. The SAVRY Total Score, SAVRY SRR, and YLS/CMI Total Score were positively and significantly correlated with violent and general recidivism. Moreover, the SAVRY Protective Total Score was negatively and significantly correlated with the SAVRY Total Score and SRR, as well as YLS/CMI Total Score and Overall Risk Rating (ORR). However, it was noted that the SAVRY Protective Total Score was negatively and significantly correlated with general but not with violent recidivism.

**Table 1:** Correlations Between SAVRY and YLS/CMI Ratings and Recidivistic Outcomes

	SAVRY Total	SAVRY SRR	SAVRY Protect. Total	YLS/CMI Total	YLS/CMI ORR	Violent Recid.	General Recid.
SAVRY Total	—						
SAVRY SRR	.75***	—					
SAVRY Protective Total	−.56***	−.42***	—				
YLS/CMI Total	.64***	.50***	−.35***	—			
YLS/CMI ORR	.47***	.42***	−.24**	.83***	—		
Violent Recidivism	.20*	.18*	−.08	.23**	.14	—	
General Recidivism	.38***	.29***	−.23**	.39***	.32***	.40***	—

*Note.* SAVRY = Structured Assessment of Violence Risk in Youth; YLS/CMI = Youth Level of Service/Case Management Inventory; “SAVRY Prot. Total” = SAVRY Protective Total Score; “Violent Recid.” = violent recidivism; “General Recid.” = general recidivism; SRR = Summary Risk Rating.

* *p* < .05. **p < .01. ***p < .001.

#### ROC Analyses

Table 2 shows the means, as well as predictive validity, for the SAVRY Total Score, SAVRY SRR, SAVRY Protective Total Score, YLS/CMI Total Score, and YLS/CMI ORR in relation to predicting recidivistic outcomes. In terms of predicting violent recidivism, the differences between the predictive validity of the SAVRY Total Score and SAVRY SRR, as well as SAVRY Total Score and YLS/CMI Total Score were nonsignificant, with analyses revealing moderate effect sizes for the prediction of violent recidivistic outcomes (AUCs = .63-.69). It was also observed that the YLS/CMI ORR was poor at predicting violent recidivism (AUC = .59).

**Table 2:** The Predictive Validity of SAVRY and YLS/CMI Ratings for Violent and General Recidivism (ROC analyses) for Entire Follow-Up Period

			Violent Recidivism		General Recidivism	
Measures	*M* (*SD*)	Range	AUC *(SE)*	95% CI	AUC *(SE)*	95% CI
SAVRY Total Score	16.78 (6.21)	2-31	.65* (.06)	[.53, .77]	.72*** (.04)	[.64, .80]
Historical	5.70 (2.90)	0-14	.66** (.06)	[.55, .78]	.70*** (.04)	[.62, .78]
Social/Contextual	5.09 (2.09)	0-11	.59 (.06)	[.48, .70]	.62** (.04)	[.53, .71]
Individual/Clinical	6.12 (2.70)	0-13	.62* (.07)	[.49, .75]	.67*** (.04)	[.59, .76]
SAVRY Protective Total^†^	2.44 (1.53)	0-6	.55 (.06)	[.43, .68]	.62** (.04)	[.54, .71]
YLS/CMI Total	14.01 (4.34)	2-24	.69** (.05)^a^	[.59, .79]	.72*** (.04)^b^	[64, .80]
Prior/Current Offenses/Dispositions	0.50 (0.75)	0-5	.58 (.06)	[.45, .70]	.60* (.04)	[.52, .69]
Family Circumstances/Parenting	2.67 (1.41)	0-6	.64* (.06)	[.53, .76]	.63** (.04)	[.54, .71]
Education/Employment	3.05 (1.65)	0-6	.63* (.06)	[.51, .74]	.63** (.04)	[.54, .71]
Peer Relations	3.42 (1.07)	0-4	.66** (.05)	[.57, .76]	.57 (.05)	[.48, .66]
Substance Abuse	0.33 (0.58)	0-2	.56 (.06)	[.44, .68]	.58 (.05)	[.49, .67]
Leisure/Recreation	1.80 (0.76)	0-3	.55 (.06)	[.43, .67]	.62* (.04)	[.53, .70]
Personality/Behavior	1.24 (1.08)	0-4	.60 (.06)	[.48, .72]	.65** (.04)	[.57, .73]
Attitudes/Orientation	1.12 (0.54)	0-3	.59 (.06)	[.47, .72]	.58 (.05)	[.49, .66]
	*n*	%				
SAVRY SRR (for violence)			.63* (.06)	[.52, .75]		
Low	45/163	27.6				
Moderate	73/163	44.8				
High	45/163	27.6				
YLS/CMI ORR			.59 (.06)^a^	[.47, .70]	.64** (.04)^b^	[.56, .73]
Low	26/163	16.0				
Moderate	119/163	73.0				
High	18/163	11.0				

*Note.* AUCs in bold denote that they are statistically significant even after False Discovery Rate corrections. AUC = Area Under Curve; SAVRY = Structured Assessment of Violence Risk in Youth; YLS/CMI = Youth Level of Service/Case Management Inventory; SRR = Summary Risk Rating.

a,b. The same alphabet denotes that the AUCs differ significantly.

† Denotes that the SAVRY Protective Total Score was used to predict desistance from recidivism instead of recidivism.

* *p* < .05. **p < .01. ***p < .001.

With regard to predicting general recidivism, the SAVRY and YLS/CMI Total Scores showed large effect sizes (AUCs = .72), and that the YLS/CMI ORR significantly predicted general recidivism with a moderate effect size (AUC = .64). However, the SAVRY ORR was poor at predicting general recidivism (AUC = .55; presumably because the ORRs were for violent and not general recidivistic outcomes). Furthermore, there were some significant differences in terms of the effect sizes for predicting general recidivism when comparing the SAVRY and YLS/CMI Total Scores versus the SAVRY SRR and YLS/CMI ORR (see Table 2). Last, the SAVRY Protective Total Score showed moderate predictive validity for desistance from general recidivism but was poor at predicting desistance from violent recidivism.

#### Cox Regression Analyses

**Figure 1:**
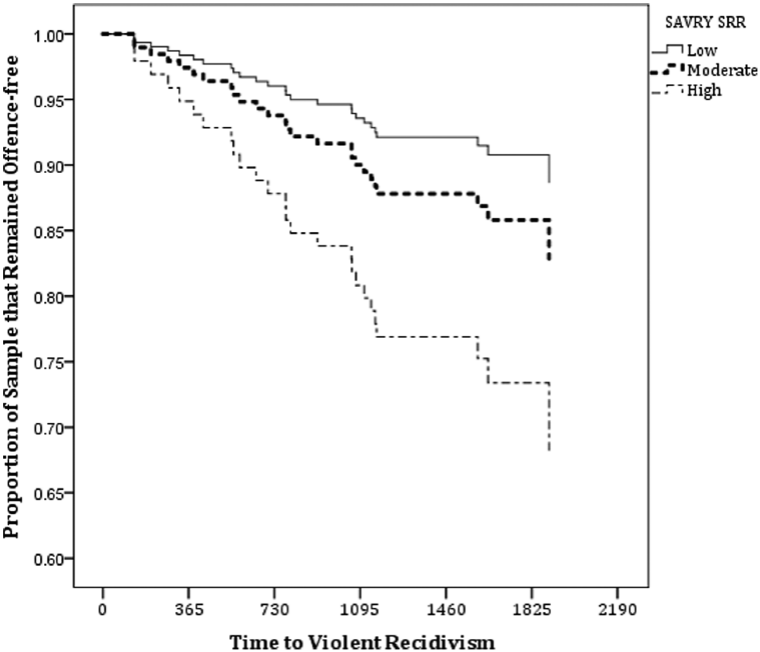
Survival Curve for SAVRY SRR and Violent Recidivism *Note.* SAVRY = Structured Assessment of Violence Risk in Youth; SRR = Summary Risk Rating.

Table 3 shows the Cox regression analyses for the SAVRY and YLS/CMI ratings in terms of predicting violent and general recidivism. The SAVRY and YLS/CMI Total Scores were significantly associated with the speed of violent and general recidivism. The SAVRY SRR classifications (see also Figure 1) consistently showed significant differences between low- and high-risk offenders in terms of their rate of violent and general recidivism, whereas the YLS/CMI ORR were only useful for classifying offenders in terms of their risk of general recidivism but not violent recidivism. The distributions of violent recidivists to SAVRY SRR classifications of low, moderate, and high were 8.9%, 13.7%, and 26.7%, respectively. Furthermore, SAVRY Protective Total Score significantly predicted general recidivism after accounting for time-at-risk but not violent recidivism.

**Table 3:** The Predictive Validity of SAVRY and YLS/CMI Ratings for Violent and General Recidivism (One-Predictor Cox Regression Models)

Measure	*B*	*SE*	Wald	HR	95% CI	*p*
Violent Recidivism						
SAVRY Total	0.09	0.03	6.15	1.09	[1.02, 1.17]	.013
SAVRY SRR						
Moderate (vs. low)	0.46	0.59	0.59	1.58	[0.49, 5.03]	.443
Moderate (vs. high)	−0.69	0.43	2.58	0.50	[0.22, 1.16]	.108
High (vs. low)	1.14	0.58	3.90	3.14	[1.01, 9.75]	.048
SAVRY Prot. Total	−0.13	0.13	1.01	0.88	[0.68, 1.14]	.316
YLS/CMI Total	0.13	0.03	22.64	1.13	[1.08, 1.19]	<.001
YLS/CMI ORR						
Moderate (vs. low)	1.55	1.02	2.30	4.73	[0.64, 35.21]	.129
Moderate (vs. high)	−0.16	0.55	0.08	0.85	[0.29, 2.50]	.774
High (vs. low)	1.71	1.12	2.33	5.54	[0.62, 49.82]	.127
General Recidivism						
SAVRY Total	0.09	0.02	25.53	1.10	[1.06, 1.14]	<.001
SAVRY SRR						
Moderate (vs. low)	0.28	0.29	0.95	1.33	[0.75, 2.35]	.331
Moderate (vs. high)	−0.80	0.24	10.83	0.45	[0.28, 0.72]	.010
High (vs. low)	1.08	0.29	13.61	2.95	[1.66, 5.23]	<.001
SAVRY Prot. Total	−0.23	0.07	10.10	.79	[0.69, 0.92]	.001
YLS/CMI Total	0.13	0.03	23.70	1.14	[1.08, 1.20]	<.001
YLS/CMI ORR						
Moderate (vs. low)	0.73	0.38	3.79	2.08	[1.00, 4.34]	.051
Moderate (vs. high)	−0.81	0.28	8.25	0.45	[0.26, 0.77]	.004
High (vs. low)	1.54	0.44	12.54	4.67	[1.99, 10.94]	<.001

*Note.* SAVRY = Structured Assessment of Violence Risk in Youth; YLS/CMI = Youth Level of Service/Case Management Inventory; SRR = Summary Risk Rating; “SAVRY Prot. Total” = SAVRY Protective Total Score; CI = confidence interval; HR = Hazard Ratio.

Table 4 shows the predictive validity of the SAVRY subscales for violent and general recidivism. None of the SAVRY subscales significantly predicted violent recidivism when entered into the Cox regression model simultaneously; however, only the Historical subscale significantly predicted general recidivism. Last, the SAVRY Protective Total Score did not add incremental predictive validity for violent and general recidivism after controlling for SAVRY Total Score in the Cox regression models (see Table 5).

**Table 4:** The Predictive Validity of the SAVRY Subscales for Violent and General Recidivism (Subscales Were Entered Simultaneously Into the Cox Regression Models)

Measure	*B*	*SE*	Wald	HR	95% CI	*p*
Violent Recidivism						
SAVRY Historical	0.11	0.07	2.62	1.12	[0.98, 1.29]	.105
SAVRY Social/Contextual	0.02	0.11	0.04	1.02	[0.83, 1.27]	.836
SAVRY Individual/Clinical	0.09	0.10	0.83	1.09	[0.90, 1.32]	.363
General Recidivism						
SAVRY Historical	0.14	0.04	12.89	1.15	[1.07, 1.25]	<.001
SAVRY Social/Contextual	−0.01	0.06	0.04	0.99	[0.88, 1.11]	.849
SAVRY Individual/Clinical	0.10	0.05	3.31	1.10	[0.99, 1.22]	.069

*Note.* SAVRY = Structured Assessment of Violence Risk in Youth; CI = confidence interval; HR = Hazard Ratio.

**Table 5:** The Predictive Validity of the SAVRY Prot. Total Score After Accounting for SAVRY Total Score (Cox Regression Model)

Measure	*B*	*SE*	Wald	HR	95% CI	*p*
Violent Recidivism						
Block 1						
SAVRY Total	0.09	0.03	6.15	1.09	[1.02, 1.17]	.013
Block 2						
SAVRY Total	0.09	0.04	5.58	1.09	[1.02, 1.18]	.018
SAVRY Prot. Total	0.05	0.16	0.08	1.05	[0.77, 1.42]	.772
General Recidivism						
Block 1						
SAVRY Total	0.09	0.02	25.53	1.10	[1.06, 1.14]	<.001
Block 2						
SAVRY Total	0.08	0.02	16.59	1.09	[1.05, 1.13]	<.001
SAVRY Prot. Total	−0.06	0.09	0.52	0.94	[0.80, 1.11]	.471

*Note*. SAVRY = Structured Assessment of Violence Risk in Youth; “SAVRY Prot. Total” = SAVRY Protective Total Score; CI = confidence interval; HR = Hazard Ratio.

## Discussion

### Predictive Validity of the Savry Ratings in a Non-Western Context

Overall, the ROC and correlational analyses (with a mean follow-up of about 4.5 years) revealed that the SAVRY Total Scores and SRR were moderately predictive of violent (AUC_Total_ = .65, *r*_Total_ = .20; AUC_SRR_ = .63, *r*_SRR_ = .18) and general recidivism for the male youth offenders (AUC_Total_ = .72, *r*_Total_ = .38; AUC_SRR_ = .65, *r*_SRR_ = .29). In addition, Cox regression analyses showed that the SAVRY Total Score was significantly predictive of time to violent and general recidivism, and that the SAVRY SRR was able to differentiate between low- and high-risk offenders well—albeit with mixed success in terms of differentiating the moderate-risk offenders from other risk groups. In addition, the SAVRY appeared to have similar predictive prowess for violent and general recidivism when compared with an established youth offender risk assessment measure, such as the YLS/CMI (violent recidivism: AUC_SAVRY_Total_ = .65 vs. AUC_YLS/CMI_Total_ = .69; general recidivism: AUC_SAVRY_Total_ = .72 vs. AUC_YLS/CMI_Total_ = .72). However, the predictive validity of the SAVRY SRR for violent recidivism was somewhat higher than the YLS/CMI ORR (AUCs = .63 vs. .59), but the SAVRY ORR’s predictive validity for general recidivism was lower than the YLS/CMI ORR (AUCs = .55 vs. .64). This smaller effect size was presumably because the SAVRY ORR pertained to the raters’ assessment of the participants’ risk of violence and not their risk of general reoffending.

The mean SAVRY Total Score for this Singaporean sample was generally lower than the Western studies (e.g., Dolan & Rennie, 2008; Gammelgård et al., 2008; Hilterman et al., 2014; Lodewijks et al., 2008; Meyers & Schmidt, 2008; Shepherd et al., 2014), but the mean SAVRY Protective Total Score was noted to be higher than most except Hilterman et al.’s results. In addition, closer examination revealed that higher Historical and Individual/Clinical subscale scores were reported in the Western studies in comparison with our present study. Similar to the SAVRY Total Score, the mean YLS/CMI Total score for this sample was lower than those obtained in the Western studies. However, the YLS/CMI Total Score was higher than those reported in Chu et al. (2015) and Takahashi et al. (2013). These observed differences could be a function of the characteristics of the comparison groups (e.g., high-risk, institutionalized youth offenders, and those with mental health issues), differences in the operationalization and enforcement of legislations, and cross-boundary differences (e.g., a relatively less serious youth crime situation in Singapore as compared with other Western contexts). Within Singapore, this present sample represented a group of youth offenders with more serious offending issues who were referred by the Probation Services Branch for psychological assessment; hence, it was expected that their YLS/CMI Total Scores would be higher than the scores reported for the general probation sample in Chu et al.

In comparison with the indices obtained from Olver et al.’s (2009) meta-analysis for non-Canadian samples, the effect sizes from the present study were somewhat lower when examining the predictive validity of the SAVRY ratings for violent recidivism, but were higher for general recidivism. Further examination revealed that the AUCs obtained for this study were somewhat lower than the Canadian studies that included at least 3 years of follow-up (see Table 6), but were similar to those from the United States and the Netherlands. Nevertheless, the base rates for violent recidivism in the Canadian samples were much higher than the present study as well as those from the United States and the Netherlands, which is a possible factor that could affect predictive validity for violent recidivism. In comparison, the effect sizes of the YLS/CMI ratings from this study were also higher than Olver et al.’s for general recidivism. Notwithstanding the significant predictive validity of the SAVRY and YLS/CMI ratings, there is still substantial variance in the violent and general recidivism rates between the offenders that is unaccounted for by the variables that were examined, and this unexplained variance could be due to differences in intraindividual-, environmental-, and system-level variables (e.g., developmental changes across the follow-up period, differences between actual and reported crimes as well as neighborhood crime rates and socioeconomic factors; Olver et al., 2009; Onifade, Petersen, Bynum, & Davidson, 2011). Nevertheless, this is the first study that had examined the predictive validity of SAVRY in the Singapore context—in comparison with a locally established youth offender risk assessment measure, YLS/CMI.

**Table 6:** Predictive Validity of SAVRY Ratings in Published Studies Across Different Countries (Nonexhaustive List)

				AUC		
Country	Study	M_follow-up_	BRVR	Violent	Nonviolent	General
Canada	Catchpole and Gretton (2003)	1 year	23%	.73		.74
	Meyers and Schmidt (2008)	1 year	16.6%	.66, .66^m^, .68^f^, .64^nc^, .65^c^	.80,	.75
		3 years	28.1%	.77, .78^m^, .80^f^, .84^nc^, .70^c^	.68	.76
	Penney, Lee, and Moretti (2010)	2 years	47%	.69^m^, .64^m,s^, .72^f^, .72^f,s^	.76^m^, .69^m,s^, .65^f^, .67^f,s^	
	Schmidt, Campbell, and Houlding (2011)	10.4 years	47.4%	.78^m^, .71^m,s^, .67^m,p^, .57^f^, .57^f,s^, .58^f,p^	.78^m^, .72^m,s^, .72^m,p^, .68^f^, .63^f,s^, .56^f,p^	.74
	Welsh, Schmidt, McKinnon, Chattha, and Meyers (2008)	35.8 months	25.7%	.81	.55	.77
The United States	Elkovitch et al. (2008)	80 months	10.2%	.63^s^		
	McGowan, Horn, and Mellott (2011)^e^	1 year	36%	.72, .66^p^		
	Viljoen et al. (2008)	6.58 years	10.1%^mv^,	.69, .59^s^, .62^p^		
			12.7%^sv^			
The United Kingdom	Dolan and Rennie (2008)	1 year	38.4%	.64, .64^s^		.69, .69^s^
	Rennie and Dolan (2010)	1 year	36.9%			.71^p^
Spain	Hilterman, Nicholls, and van Nieuwenhuizen (2014)	1 year	65.4%	.75, .68^s^, .63^p^		.71, .51^p^
The Netherlands	Lodewijks, Doreleijers, de Ruiter, and Borum (2008)^r^	22 months	—	.80, .86^s^, .87^p^		
	Lodewijks et al. (2008)	3 years	19.7%	.65, .71^s^, .72^p^		
Finland	Gammelgård et al. (2008)	6 months	23.1%	.71		
Australia	Shepherd, Luebbers, Ferguson, Ogloff, and Dolan (2014)	6-18 months	59%	.66, .64^s^, .68^es^, .66^es,s^, .77^es,p^, .47^cld^, .48^cld,s^, .57^cld,p^, .76^ind^, .74^ind,s^, .67^ind,p^		.70, .68^s^, .78^es^, .76^es,s^, .80^es,p^, .49^cld^, .50^cld,s^, .57^cld,p^, .81^ind^, .74^ind,s^, .91^ind,p^

*Note.* “Violent,” “nonviolent,” and “general” refer to violent, nonviolent, and general recidivism, respectively, and all AUCs pertain to total sample and SAVRY total score unless stated otherwise. SAVRY = Structured Assessment of Violence Risk in Youth; BRVR = base rate of violent recidivism; AUC = Area Under Curve.

m,f. Refer to male and female, respectively.

nc,c,es,cld,ind. Refer to native Canadian, Caucasian, English-speaking background, culturally and language diverse background, and Indigenous background, respectively.

s,p. Refer to summary risk rating and protective factor domain, respectively.

e,r. Refer to a strictly education and residential sample, respectively.

mv,sv. Refer to minor violence and serious violence, respectively.

Apropos of comparing the SAVRY and YLS/CMI Total Scores against the SAVRY SRR and YLS/CMI ORR, it appeared the AUCs of the total scores for violent and general recidivism were better than the omnibus ratings (i.e., SRR and ORR). Cox regression analyses showed similar results, and closer examination suggests that the SAVRY SRR was only somewhat useful for predicting violent recidivism. The YLS/CMI ORR was poor in discriminating the violent recidivists from nonrecidivists after accounting for time, but was good for predicting general recidivism. Apart from the SAVRY Historical subscale that showed significant predictive validity for general recidivism, the rest of the SAVRY subscales did not predict violent and general recidivism when entered simultaneously into Cox regression models. These results suggest that the dynamic risk factors may contribute less than the static risk factors over longer term (Chu, Thomas, Ogloff, & Daffern, 2013), but this is an area that warrants more empirical investigation. With regard to the distribution of violent recidivists to SRR, 26.7% and 13.7% of the high-risk and moderate-risk group reoffended violently, respectively. When compared with existing literature (e.g., Meyers & Schmidt, 2008), these figures seemed low—about half the rate over a similar follow-up period. However, it is important to examine these recidivism rates in the context of low crime and recidivism rates within the Singaporean context. For example, the 3-year recidivism rate for youth offenders placed on community supervision is usually around 10%.

With regard to the SAVRY Protective Total Score, the present findings showed that it was only useful for predicting desistance from general recidivism (albeit a relatively modest effect size), and not violent recidivism. Correlational, Cox regression, and ROC analyses consistently revealed that there was a lack of relationship between SAVRY Protective Total Score and violent recidivism. This finding could, in part, explain the lower predictive validity of the SAVRY SRR (as compared with SAVRY Total Score) for both violent and general recidivism because the raters took into account the protective factors when making SRRs. More importantly, the present results were somewhat inconsistent with existing findings (e.g., Hilterman et al., 2014; Lodewijks, de Ruiter, & Doreleijers, 2010; Schmidt et al., 2011), which showed that the SAVRY Protective Total Score has significant predictive validity for desistance from violent recidivism. However, Shepherd et al. (2014) found that ethnic and cultural differences could affect the predictive validity of protective factors for desistance from violent recidivism, and Shepherd et al.’s and the present findings collectively suggest that there is a need to reexamine the protective factors that buffer youth against violent recidivism when the SAVRY is used cross-culturally. Last, the SAVRY Protective Total Score did not show incremental predictive validity for violent and general recidivism over the SAVRY Total Score, suggesting that the protective factors did not contribute incremental information over and above the baseline risk.

Overall, it is arguable whether the SAVRY should be used to predict violence in Singaporean youth offenders. At the moment, the present findings suggest that there was no compelling advantage to use the SAVRY over the YLS/CMI when predicting violent and general recidivism in the absence of further data. Moreover, the SAVRY protective factors did not offer a lot in terms of predicting desistance to recidivism. Nevertheless, it is understandable that clinicians might prefer to use the SAVRY for case formulation, and ultimately violence prevention, due to the inclusion of many purported, violence-specific criminogenic risk factors/needs.

### Limitations and Future Directions

First, electronic data and archival file data were used to code the SAVRY and recidivism follow-up; thus, it is likely that there would be an underestimate of the reoffending due to the further offenses not having been detected. Moreover, it is unclear whether some risk and protective factors should be equally weighted when we consider these variables in determining risk; these issues need to be explored further. We also did not check the reliability and validity of the SRR from file information—this should be addressed in future studies. Furthermore, we did not compare the predictive validity of the ratings that were completed by the clinicians and researchers. It would be important to clarify whether there are significant differences in future studies.

Future research on the SAVRY should use prospective and repeated-measures designs, in which the SAVRY ratings are based on interviews as well as available information from archival records. Moreover, it is beneficial to examine gender differences with regard to the usage of the SAVRY in the local context, so that the necessary adaptation can be made. There was a large proportion of the high-risk youth offenders who did not reoffend violently; as such, the predictive accuracy of the SAVRY would be improved if research can identify which factors (or combination) can characterize the violent recidivists better. In addition, it might also be helpful to identify what a specific score from the SAVRY means in relation to another score from the YLS/CMI when we examine this issue through a risk communication perspective. Last, future research can focus on identifying the relevant protective factors for desistance from violent recidivism in non-Western contexts, and also to clarify the pathways through which the protective factors operate.

## Conclusion

Notwithstanding the limitations, the present findings suggest that there is value in using the SAVRY for assessing the risk of violent and general recidivism in youth offenders within the Singaporean context. Broadly speaking, the results of this study are generally comparable with other studies from non-Canadian jurisdictions despite some differences, and provide evidence that the SAVRY ratings maintain their predictive validity for at least 3 years (with stronger results observed in the second- and third-year of follow-up). Interestingly, the present study showed that the SAVRY risk and protective ratings are able to predict general recidivism better than violent recidivism; this suggests that many items on the SAVRY tap into the larger construct of general recidivism and that there is a big overlap in risk factors for violent recidivism and general antisociality. Risk and need assessment measures are best used for preventive purposes to classify target groups, as well as to guide and allocate resources for intervention. In addition, they can be used to encourage the youth offender to work toward the development of prosocial trajectories. Pertaining to these aspects, this study demonstrated that the SAVRY offers promise for such purposes in Singapore to varying extents.

## Notes

1. AUC_Violence_ refers to the Area Under Curve for predicting violence-related outcomes.

2. AUC_GR_ and AUC_VR_ refer to the Area Under Curve for predicting general recidivism and violent recidivism, respectively.

3. AUC_NVR_ refers to the Area Under Curve for predicting nonviolent recidivism.

4. These youth offenders represented approximately 12% to 16% of the youth offender population (aged 12 to 18 years old).

5. The differences in the denominators are due to missing data.

6. The differences in terms of age at referral, *t*(161) = 0.04, *p* = .97, age at first charged offense, *t*(151) = −1.81, *p* = .07, education level, intact family of origin, the total number of index offenses, *t*(153) = .90, *p* = .93, and the number of violent, *t*(153) = .53, *p* = .60, and sexual, *t*(158) = −.73, *p* = .47, index offenses between the two groups were nonsignificant in nature; thus, we had examined the sample as a whole in our analytical strategy.

7. Sexual offenses refer attempted rape, indecent exposure, molestation, peeping, rape, and sodomy. This rate of sexual offending is not representative of the rate of sexual offending for young offenders in Singapore as the assessment service sees an over-representation on youth charged with sex offenses.

8. Theft/fraud offenses refer to all forms of theft, criminal breach of trust (to cheat others), embezzlement, and possession and handling of stolen goods or proceeds of crime.

9. Substance use offenses refer to consumption of illicit drugs (e.g., amphetamines cannabis, cocaine, heroin, and other hallucinogenic drugs), illicit use of inhalants (e.g., glue, paint, and petrol), underage consumption of alcohol, and the unauthorized use of prescription drugs.
